# Active Stand Testing for Identification of Postural Orthostatic Tachycardia Syndrome

**DOI:** 10.7759/cureus.38281

**Published:** 2023-04-29

**Authors:** Deirdre Breslin, Pádraig Synnott, Colm Byrne

**Affiliations:** 1 Emergency Medicine, Mater Misericordiae University Hospital, Dublin, IRL; 2 Geriatrics, Mater Misericordiae University Hospital, Dublin, IRL

**Keywords:** finometer, comprehensive geriatric assessment, active stand, dizziness, postural orthostatic tachycardia syndrome (pots)

## Abstract

A 67-year-old woman presented to the Emergency Department (ED) of a Level 4 Hospital with a history of “dizziness” on standing. The front-door frailty team, including a consultant geriatrician, reviewed her in the ED. An Active Stand test was performed on the day of attendance, identifying Postural Orthostatic Tachycardia Syndrome (POTS). This was attributed to underlying adrenal insufficiency and managed with oral steroids.

“Dizziness” is a common presentation to Emergency Departments and can be challenging to investigate. This case report describes the application of Comprehensive Geriatric Assessment, the novel use of beat-to-beat non-invasive blood pressure monitoring in an Emergency Department setting, and the benefits for an individual patient presenting with a complaint of “dizziness”. While the benefits of Comprehensive Geriatric Assessment have previously been described, our case report suggests that Active Stand testing in an ED setting may help clarify this presentation. Further research in this area could prove beneficial to patients.

## Introduction

Dizziness is one of the most common presenting complaints in Emergency Departments [1], and it can be challenging to investigate and manage. The term is somewhat nebulous, and self-reported “dizziness” may reflect various symptoms, including pre-syncope, vertigo, unsteadiness, and malaise [2]. Thorough history taking and physical examination are the cornerstones of assessing this complaint, but the appropriate investigation may bolster these. This case demonstrates a case of sub-acute dizziness, where a diagnosis was reached through Active Stand testing in the ED.

## Case presentation

A 67-year-old woman presented to the Emergency Department with a history of “dizziness and unsteadiness on [her] feet”, present for almost a year but worsening in the last month. She described “dizziness” when moving from lying/sitting to standing or when standing still for a few minutes. She also reported feeling internally “shaky” and unwell during these episodes, though no objective tremor had been observed. She had no history of falls or syncopal episodes.

Her medical history was significant for cryoglobulinemia with prior rituximab treatment, rheumatoid arthritis, bilateral knee replacements, and recurrent leg ulcers. Her pre-attendance medications were prednisolone 5mg and Vitamin D replacement. Her Clinical Frailty Score was 4.

**Figure 1:**
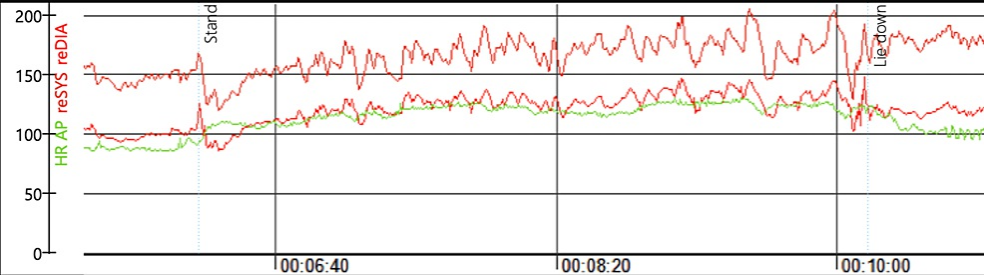
Heart rate (HR) response to standing - green line. HR rose from approximately 90bpm to over 120bpm for >3 minutes in response to active stand.

As part of her Emergency Department work, a Comprehensive Geriatric Assessment was performed by members of the hospital’s front-door frailty team - in this case, a Specialist Registrar and a Consultant in Medicine for the Older Person. An Active Stand test was performed, using beat-to-beat non-invasive blood pressure measurement with a Finometer, and this test did not demonstrate a significant blood pressure drop. However, sustained tachycardia was elicited, with the patient’s heart rate rising > 30 bpm on moving from lying to standing. This tachycardia persisted for over 3 minutes until the patient lay back down and was associated with reproducing her “dizzy” symptoms. Figure 1 below is the recorded BP and Heart rate (HR) trace.

In conjunction with the duration of symptoms, this Active Stand picture can suggest a diagnosis of Postural Orthostatic Tachycardia Syndrome (POTS). It was, therefore, important to assess if there was any secondary cause for the patient’s excessive tachycardia response. On review of the patient’s medical history and co-morbidities, this orthostatic tachycardia was attributed to adrenal insufficiency secondary to chronic steroid use. The patient’s regular steroid dose was increased from 5mg to 7.5mg. On review 3 weeks post-presentation, her orthostatic symptoms had fully resolved.

## Discussion

Previous studies have explored the importance of Comprehensive Geriatric Assessment (CGA) for frail and pre-frail older patients in hospital settings. Benefits for patients may include improved functional ability, lower mortality risk, and an increased likelihood of discharge to their own home following hospital admission [3]. Similarly, implementing CGA in the Emergency Department may result in more patient-centered care and tailored investigations, reducing ED re-attendance and admission rates [4]. Completing an Emergency Department CGA and early review by a consultant geriatrician led to a prompt diagnosis through an Active Stand test. 

An Active Stand test may be performed in the hospital or community setting. Within the hospital setting, the use of beat-to-beat blood pressure and heart rate measurement allows for the detection of transient cardiovascular responses, which may not be captured by traditional blood pressure monitoring [5]. This can be achieved non-invasively, using devices such as the Finometer, as in this case. Active Stand testing can elicit a variety of abnormal hemodynamic responses, such as initial orthostatic hypotension (IOH), classical orthostatic hypotension (OH), orthostatic hypertension (OHTN), and postural orthostatic tachycardia syndrome (POTS) [6]. However, it is important to acknowledge that the test has some limitations. Notably, many factors can affect the patient’s heart rate and blood pressure response, including their fasting status, recent exercise, and pre-test intake of fluids, nicotine, and caffeine [5]. Additionally, there is relatively low “test-retest reliability” observed in active stand testing, which limits its utility to track illness over long periods [5].

In this case, our patient’s Active Stand demonstrated a sustained tachycardia >30bpm without significant blood pressure change within the first 10 minutes of moving from supine to upright. This was associated with reproducing her presenting symptoms (i.e., she described “dizziness”). This pattern is consistent with a diagnosis of POTS. Once this hemodynamic pattern is identified on active stand testing, it is vital to consider secondary causes for orthostatic tachycardia. Such underlying causes may be treated with targeted management. Secondary causes of orthostatic tachycardia include but are not limited to, drugs (prescribed or recreational), endocrine disorder (e.g., adrenal insufficiency, Cushing syndrome, hyperthyroidism, pheochromocytoma), anemia and prolonged bed rest [7,8].

In our patient’s case, the orthostatic tachycardia was attributed to adrenal insufficiency and was managed by increasing the patient’s baseline steroid regimen. In the absence of underlying triggers and symptoms lasting at least 3 months, a diagnosis of primary POTS may be made, and a second active stand test should confirm the diagnosis [9]. Management of primary POTS is currently targeted toward patient education, physical therapy and conditioning, and symptom minimization strategies [9]. Non-pharmacological interventions include: increased fluid and salt intake, waist-high compression garments, and increased aerobic conditioning exercises [8]. Pharmacological interventions include the use of Propanolol, Midodrine, or Ivabradine. However, it is important to note that a paucity of robust research data exists to guide the medical management of POTS symptoms [8].

## Conclusions

“Dizziness” is a common presentation to Emergency Departments. Our case demonstrates one potential cause for this symptom and illustrates the benefit of Comprehensive Geriatric Assessment in the ED setting. It further demonstrates the role of specialized investigations, such as Active Stand testing, in assessing patients with this complaint in ED.
